# Multivariate analysis of activated sludge community in full-scale wastewater treatment plants

**DOI:** 10.1007/s11356-020-10684-5

**Published:** 2020-09-12

**Authors:** Mateusz Sobczyk, Agnieszka Pajdak-Stós, Edyta Fiałkowska, Łukasz Sobczyk, Janusz Fyda

**Affiliations:** Institute of Environmental Sciences, Jagiellonian University, Gronostajowa 7, 30-387 Krakow, Poland

**Keywords:** Activated sludge, Protozoa, Metazoa, Process parameters, PCA, RDA

## Abstract

We investigated changes in protozoa and metazoa community in relation to process parameters in activated sludge from four wastewater treatment plants (WWTPs) throughout the period of 1 year. Principal component analysis (PCA) showed that activated sludge from investigated treatment plants had different dominating species representatives and community composition mainly depends on individual features of the treatment plants. Redundancy analysis (RDA) showed that the temperature in bioreactors was the most relevant factor explaining changes in the microorganism community, whereas reduction rate of chemical oxygen demand (COD), biological oxygen demand (BOD_5_), suspended solids (SS), and total nitrogen (TN) did not sufficiently explain the variation in protozoa and metazoan community composition. The results indicate that in stable working WWTP it is difficult to find a pronounced link between activated sludge species composition, process parameters, and plant configuration. Applied multivariate analysis can be a valuable tool for the exploration of the relations between community composition and WWTP process parameters.

**Electronic supplementary material:**

The online version of this article (10.1007/s11356-020-10684-5) contains supplementary material, which is available to authorized users.

## Introduction

Curds and Cockburn (1970) were probably the first researchers who used the protozoa community as bio-indicators of effluent quality of activated sludge system plants. Later, many attempts were made to relate the physical–chemical parameters of effluent or activated sludge with the present species of ciliates and other protozoa (Morishita 1976; Madoni and Ghetti 1981; Al-Shahwani and Horan 1991; Esteban et al. 1991; Salvadó et al. 1995; Perez-Uz et al. 2010; Hu et al. 2013a). The general conclusion from these researches indicates that each wastewater treatment plant (WWTP) develops its own distinctive protozoan community which depends on the specific features of the plant itself (Seviour and Nielsen 2010) and no general and clear pattern exists. Attempt to explain this phenomenon was described by Salvadó et al. (1995), but statistical analyses used to relate physical–chemical parameters and protozoa showed that relation between various physical–chemical parameters and a particular species does not follow a linear model.

Long-term monitoring of the protozoa and metazoa community inhabiting activated sludge has already been conducted by scientists in several countries around the world: in Spain by Salvadó and Gracia (1993) and Martin-Cereceda et al*.* (1996), in Germany by Ettl (2001), in Austria by Foissner and Berger (1996), and in China by Zhou et al*.* (2006, 2008), Liu et al*.* (2008), and Hu et al*.* (2013a,b). However, results from most studies cannot be directly extrapolated to modern WWTPs designed for biological nutrient removal, and thus, new researches concerning bio-indicators are required (Perez-Uz et al*.* 2010; Dubber and Gray 2011). Recently Hu et al. (2013a,b), Zornoza (2017) observed and analyzed protozoa and metazoa community in a new types of WWTPs. Their study shows that some protozoa and metazoa representatives were related with the activated sludge system performance, particularly with effective nitrification process.

The results from four full-scale WWTPs presented in this study fit very well to the current demand of biological analysis of activated sludge and are intended to draw attention to the deficiencies of the used methods. Throughout 1 year, the changes in protozoa and metazoa community composition in relation to changes in operational or environmental parameters were studied. Using of protozoa and metazoa as bioindicators of biological oxygen demand (BOD_5_), chemical oxygen demand (COD), and of the effectiveness of the suspended solids (SS) and nitrogen (TN) removal process was evaluated.

## Materials and methods

Samples were collected from four treatment plants operating in the Małopolska voivodship, southern Poland (Table 1). For the analysis 81 samples of activated sludge were taken from the WWTP aeration tanks.

**Table 1:** Main features of investigated WWTPs

WWTP code	Technology	Real size (people equivalent)	Mean sewage flow (m^3^/day)	Working biological tank volume (m^3^)	Industrial waste (%)	N and P elimination
SK	A2/O	100,569	6387	6196 + 3698	30	Yes
NP	A2/O	82,047	5269	4998 ∗ 2	35–40	Yes
CH	MLE	73,915	15,142	2849 ∗ 2	15–20	Yes
SI	A2/O	7462	923	1940	0	No

*A2/O* anaerobic/anoxic/oxic, *MLE* modified Ludzack–Ettinger process

### Influent, effluent quality and process parameters

All investigated objects are municipal WWTPs. Plants SK, NP, and CH additionally purify the variable volume of industrial sewage inflow. In all WWTPs phosphorous was reduced mainly by chemical precipitation (e.g., PIX dosing) so its values are not included in the data analysis.

Chemical analyses of influent and effluent parameters: SS, COD, BOD_5_, TN, and TP, were carried out by SK, CH, and SI WWTP laboratories or, as in the case of NP plant by the accredited laboratory of Municipal Water and Sewage Company in Kraków. The plants’ operators provided information about operating parameters such as mixed liquor suspended solids (MLSS), hydraulic retention time (HRT), sludge load (F/M), temperature (T), and the sludge volume index (SVI) of the mixed liquor. Sludge age was not calculated by operators in the investigated plants.

### Microscopic observation

Microscopic analysis was conducted using the Nikon Eclipse 80i and Olympus IX 71 microscopes with × 200 and × 400 magnification. The density of protozoa and metazoa community was determined based on analysis of two or three 25 μl drops taken from a well-mixed activated sludge sample, immediately after delivery to the laboratory. Small flagellates were counted along the diagonal in the Fuchs-Rosenthal chamber. The microbial density was averaged and recalculated for 1 ml volume of activated sludge. Ciliate species determination was done based on Foissner et al. (1991, 1992, 1994, 1995, 1996) identification keys.

For data analysis protozoa and metazoa species were assigned to crawling, attached, swimming and predatory ciliates, naked and testate amoebas, metazoa, and flagellates. Rotifers, tardigrades, nematodes, and gastrotrichs were included into metazoa group. Flagellates consisted of two groups highlighted by Salvadó (1994), one group consisted of heterotrophic flagellates smaller than 20 μm, and the second of flagellates bigger than 20 μm.

### Statistics

Principle component analysis (PCA) and redundancy analysis (RDA) were carried out on log-transformed densities of investigated protozoa and metazoa individuals. Response data were centered and standardized by species. PCA scores obtained for the first and second axes were used as the response variable in analysis of variance (ANOVA) and to check differences in species composition and process parameters between investigated treatment plants. The Tukey test was used as post hoc test. All statistical analyses were carried out in Canoco 5 (Ter Braak and Šmilauer 2012) and Statistica 13 (TIBCO Software Inc. 2017).

## Results

### Process parameters

The investigated WWTPs differed in process values (Table 2) and parameters (Table 3). The most pronounced differences between WWTPs were observed in TN and TP reduction rate.

**Table 2:** Values of process parameters in investigated WWTPs. Mean ± standard deviation (SD)

WWTP	MLSS (g/L)	T (°C)	HRT (days)	F/M (gBOD_5_/gMLSS/day)	SVI
SK	4.73 ± 0.64	17.7 ± 2.3	1.18 ± 0.18	0.09 ± 0.04	139.3 ± 27.6
NP	4.19 ± 0.54	18.4 ± 3.6	1.65 ± 0.15	0.14 ± 0.08	128.6 ± 36.1
CH	5.00 ± 0.77	12.5 ± 3.9	0.37 ± 0.09	0.07 ± 0.05	170.6 ± 30.4
SI	4.20 ± 0.87	12.8 ± 4.7	4.37 ± 0.81	0.01 ± 0.006	194.9 ± 34.2

**Table 3:** Chemical parameters of the influent, effluent, and reduction rate in investigated WWTPs (mean ± SD)

WWTP		SS	BOD_5_	COD	TN	TP
SK	Influent (mg/l)	889 ± 487	872 ± 264	1600 ± 342	133 ± 33.8	18.2 ± 7.9
	Effluent (mg/l)	7 ± 3	6 ± 2	59 ± 14	10.6 ± 1.7	0.7 ± 0.9
	Reduction (%)	99.1 ± 0.6	99.3 ± 0.3	96.1 ± 1.2	91.4 ± 2.7	95.1 ± 7.5
NP	Influent (mg/l)	593 ± 476	934 ± 489	2151 ± 1178	101.6 ± 33.8	12.7 ± 5.5
	Effluent (mg/l)	10 ± 7	6 ± 4	41 ± 13	13.6 ± 7.7	0.8 ± 2.1
	Reduction (%)	97.9 ± 1.6	99.3 ± 0.4	97.6 ± 1.3	85.7 ± 7.5	93.7 ± 16.2
CH	Influent (mg/l)	292 ± 106	293 ± 119	630 ± 197	49.2 ± 11.1	8.8 ± 4.0
	Effluent (mg/l)	10 ± 10	8 ± 7	24 ± 9	10.8 ± 8.6	0.8 ± 0.5
	Reduction (%)	96.2 ± 3.3	97.2 ± 2.5	95.7 ± 2.0	77.2 ± 17.7	89.9 ± 5.6
SI	Influent (mg/l)	546 ± 221	492 ± 204	1001 ± 372	89.5 ± 20.82	10.9 ± 2.6
	Effluent (mg/l)	4 ± 4	5 ± 4	32 ± 9	16.9 ± 2.9	4.5 ± 2.2
	Reduction (%)	99.1 ± 0.7	98.8 ± 0.7	96.5 ± 1.4	80.4 ± 4.4	54.2 ± 28.4

**Fig. 1:**
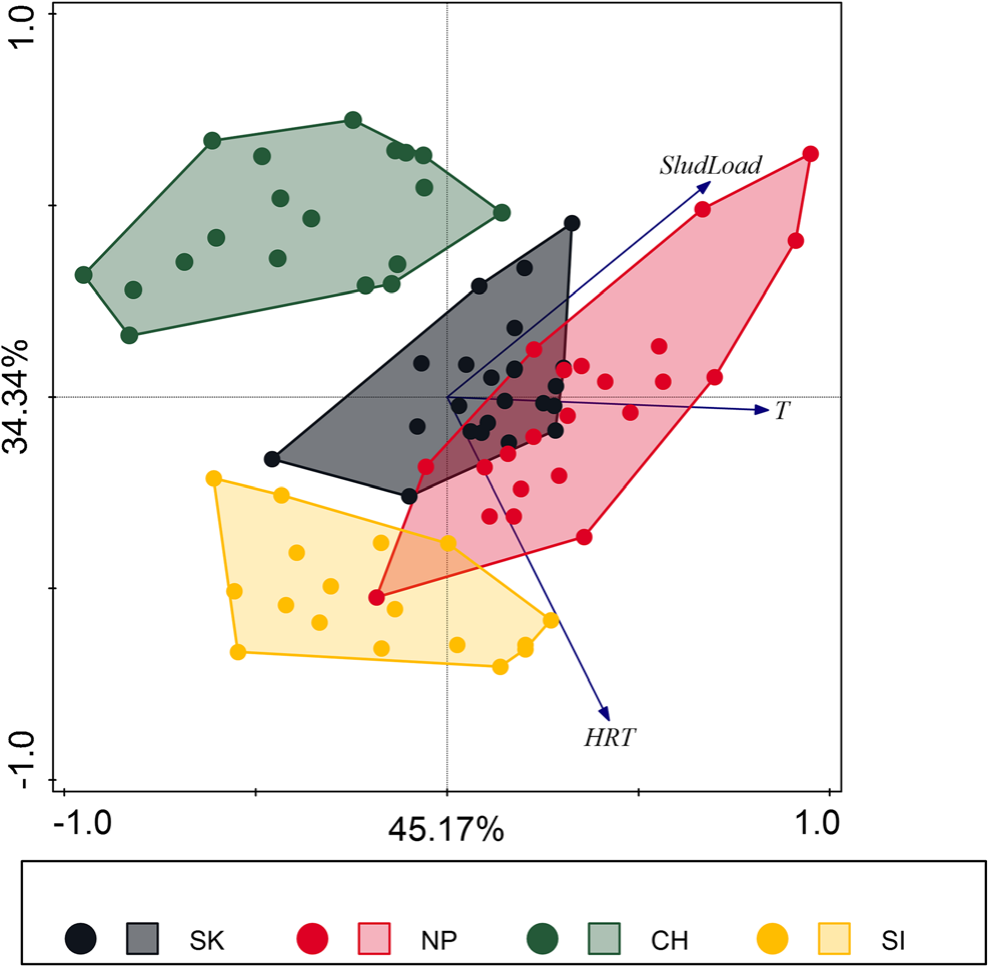
PCA analysis of process parameters of all data received during the monitoring. Arrows represent process parameters. Dots represent WWTP: black—SK, red—NP, green—CH, and yellow—SI. The first axis explained 45.17% and the second axis 34.34% of the variance in process parameters

PCA analysis (Fig. 1) showed that due to the analyzed process parameters, monitored WWTPs formed three separated groups along first PCA axis: SK WWTP, NP WWTP, and CH WWTP with SI WWTP and three separated groups along second PCA axis: SK with NP WWTP, CH WWTP, and SI WWTP. ANOVA analysis on first and second axis PCA scores showed that investigated WWTPs differ in process parameters (Axis 1: *F*_(3,77)_ = 30.17, *p* < 0.001 and Axis 2: *F*_(3,77)_ = 72.82, *p* < 0.001). PCA diagram (Fig. 1) explained 79.51% of the variance in process parameters values.

### Functional group composition

**Fig. 2:**
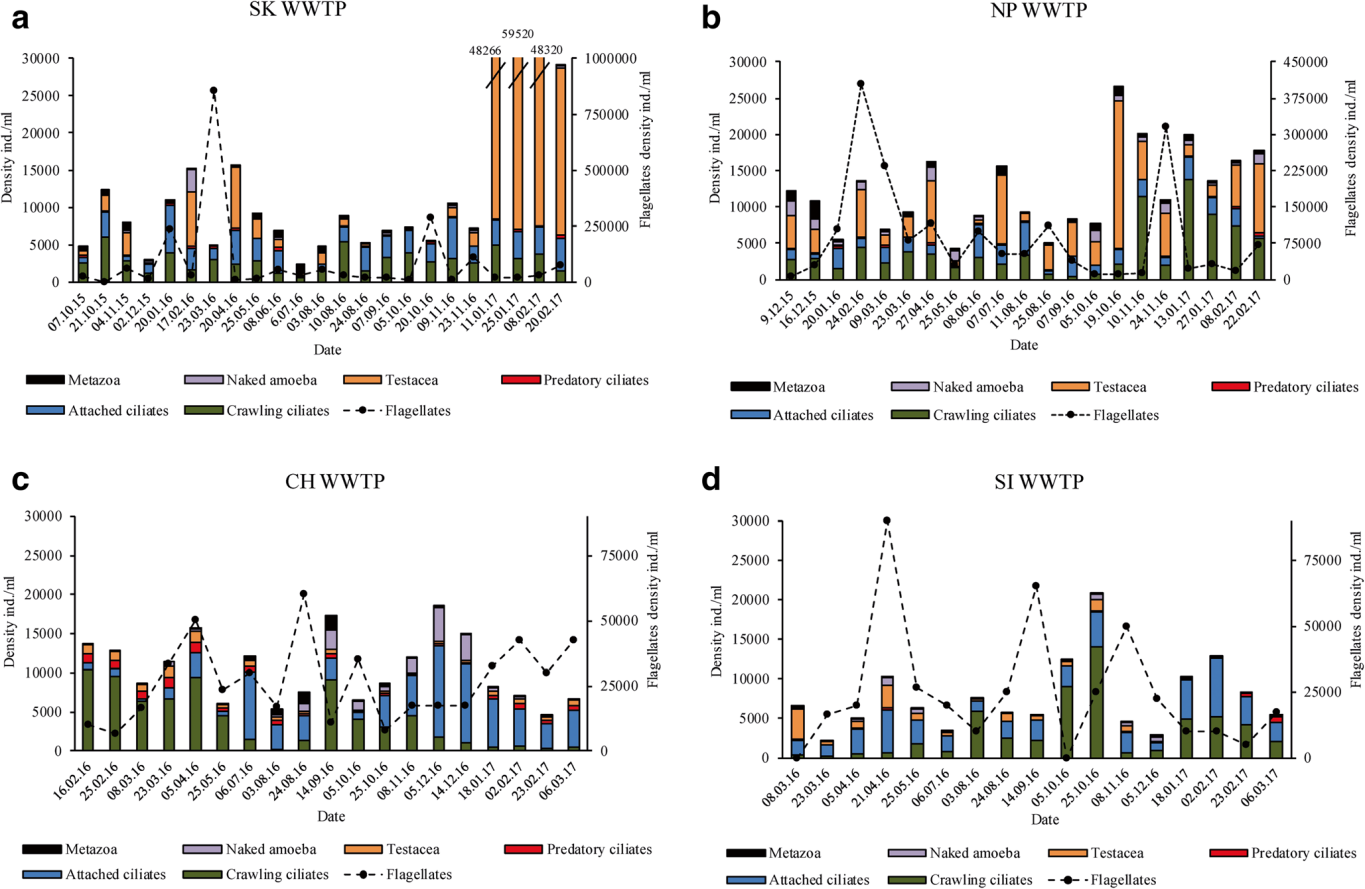
Annual variations of the density of functional groups in SK WWTP (**a**), annual variations of the density of functional groups in NP WWTP (**b**), annual variations of the density of functional groups in CH WWTP (**c**), and annual variations of the density of functional groups in SI WWTP (**d**)

During the study 34 ciliated protozoa assigned to functional groups were found. The list of protozoa and metazoa observed is included in supplementary materials (Table S1). In each of the investigated WWTPs, high fluctuations in time among protozoa and metazoa density and species composition were noticed (Fig. 2a, b, c, d). The fluctuations in protozoa and metazoa density were higher in bigger WWTPs (SK and NP) than in smaller ones (CH and SI). The most stable functional groups (with the lowest coefficient of variation CV value) were different in each WWTP during the monitoring period. In SK plant it was crawling ciliates (CV = 0.49), in NP predatory ciliates (CV = 0.53), in CH flagellates (CV = 0.57), and in SI attached ciliates (CV = 0.55). The higher density of flagellates and testate amoebae were observed in bigger WWTPs (SK and NP) then in CH and SI. In SK treatment plant during winter months in 2017 high peak of testate amoebae, represented mainly by *Cochlipodium* sp., was observed (Fig. 2a). Crawling and attached ciliates were the most numerous functional groups in all WWTPs. The least numerous of all functional groups in all treatment plants were swimming ciliates.

### Functional group composition in investigated WWTPs

**Fig. 3:**
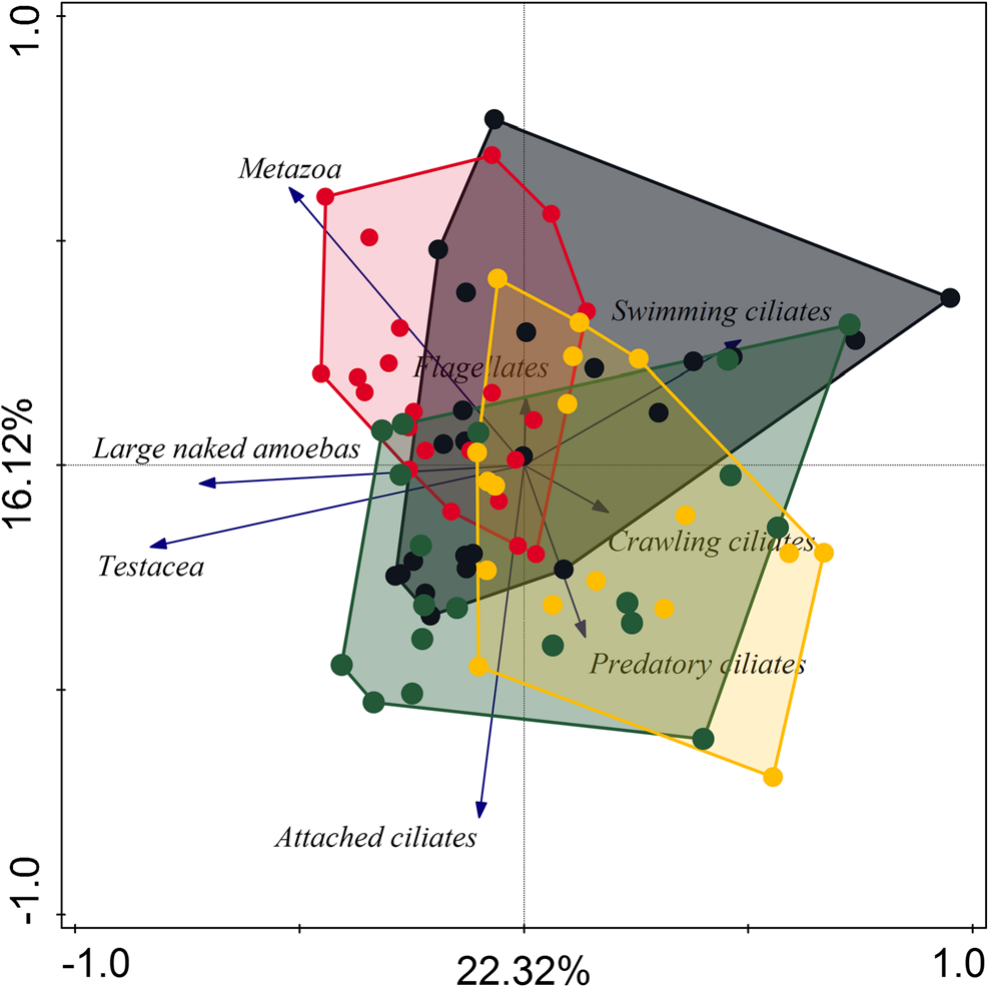
PCA analysis of the functional group composition of all data received during the monitoring. Dots represent WWTP: black—SK, red—NP, green—CH, and yellow—SI. The first axis explained 22.32% and the second axis 16.12% of the variance in activated sludge functional group community

The PCA biplot (Fig. 3) showed that none of the monitored WWTPs significantly differed from others in the composition of functional groups. Only one sample from SK and one sample from SI differed strongly from the remaining samples in functional group composition. Non-parametric Kruskal–Wallis test on first and second axis PCA scores showed that investigated WWTPs did not differ in functional group composition. ANOVA analysis was not performed because the assumption of homogeneity of variance and normal distribution of residuals were not met.

### Species composition in investigated WWTP

**Fig. 4:**
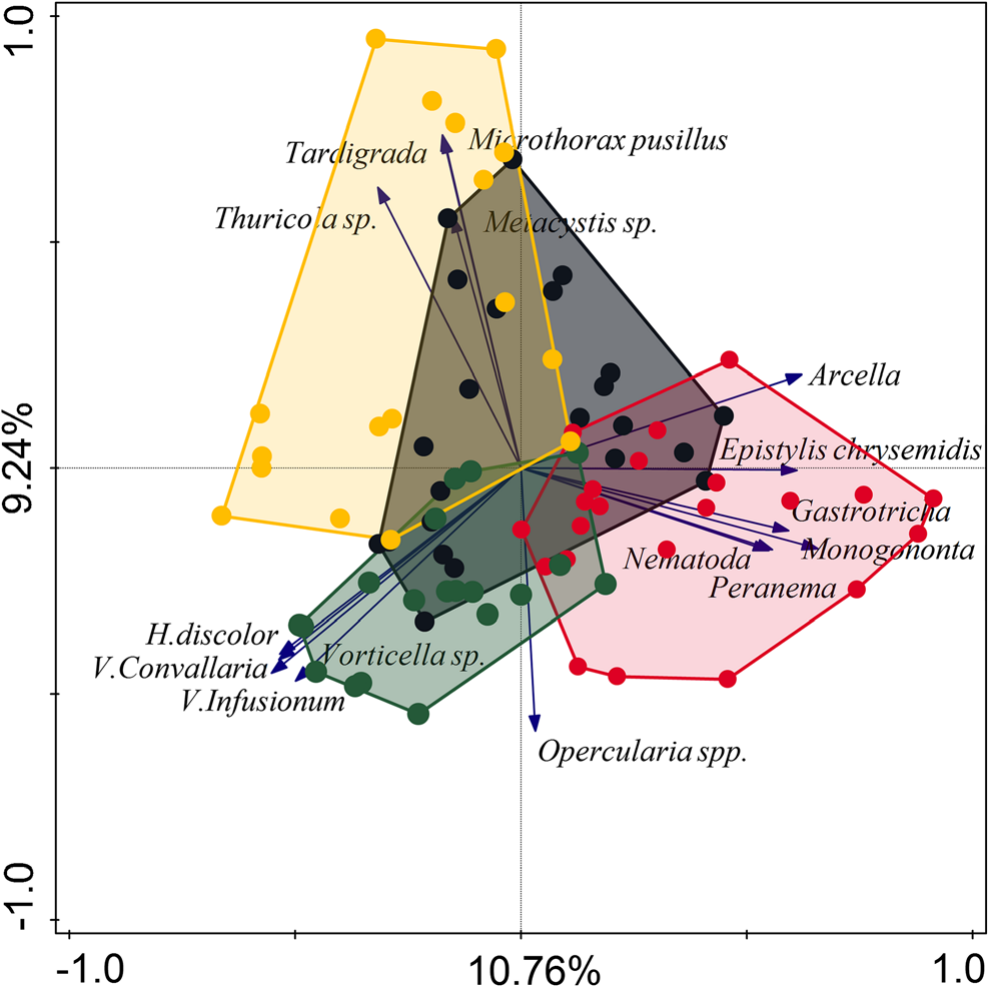
PCA analysis of activated sludge biocenosis composition of all data received during the monitoring. Arrows represent 15 best fitted protozoa and metazoa representatives. Dots represent WWTP: black—SK, red—NP, green—CH, and yellow—SI. The first axis explained 10.76% and the second axis 9.24% of the variance in protozoa and metazoa activated sludge community

**Fig. 5:**
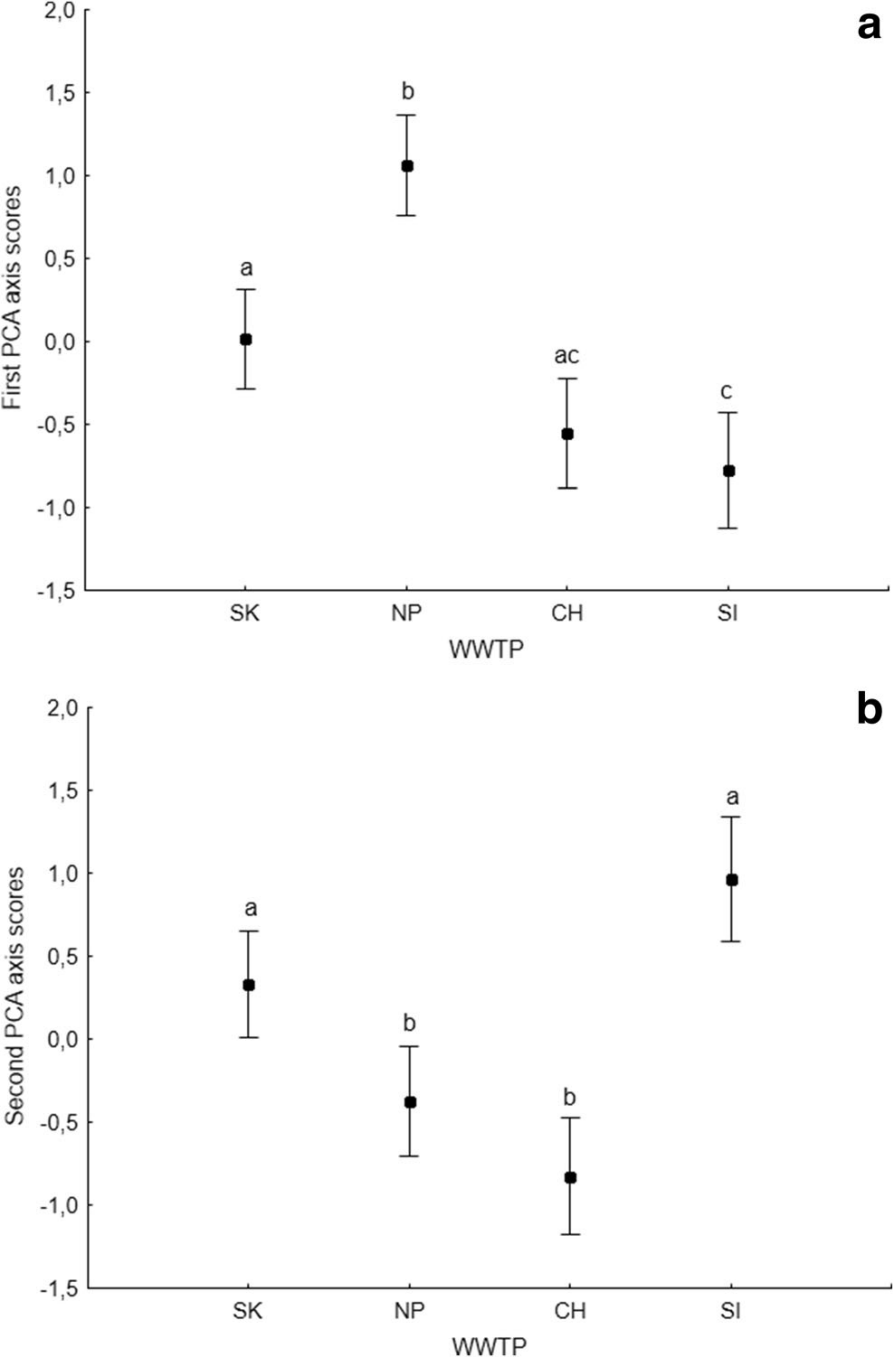
Differences in protozoa and metazoa community between investigated WWTPs based on first (**a**) and second PCA axis scores (**b**). Vertical bars denote 95% confidence intervals.

ANOVA analysis performed on first and second PCA axis scores showed that investigated WWTPs differ in assemblage of protozoa and metazoa species (Axis 1: *F*_(3,77)_ = 26.37, *p* < 0.001 and Axis 2: *F*_(3,77)_ = 19.05, *p* < 0.001) (Fig. 4). The significant differences in protozoa and metazoa communities observed between WWTPs are presented in Fig. 5 a and b. Based on the results of this analysis specific dominated representatives of protozoa and metazoa for individual WWTP could be defined. During the monitoring period, based on the first PCA axis scores NP WWTP differs significantly from other WWTPs. In NP WWTP dominated *Arcella* sp., *Peranema* sp., *Epistylis chrysemidis*, and metazoa such as gastrotrichs, nematodes, and monogononta rotifers. SK WWTP had a similar community to CH WWTP, and SI WWTP had similar protozoa and metazoa assemblage to CH WWTP (Fig. 5a). Along the second PCA axis, WWTPs formed two groups: first SK with SI where the density of *Microthorax pusillus*, *Metacystis* sp., *Thuricola* sp., and tardigrades tend to be higher and second NP with CH where occurrence of *Opercularia* spp. were higher (Figs. 4 and 5b).

However, it should be considered that PCA diagram (Fig. 4) explained only 20.00% of the variance in protozoa and metazoa species composition.

### Functional groups composition explained by process and operational parameters

**Fig. 6:**
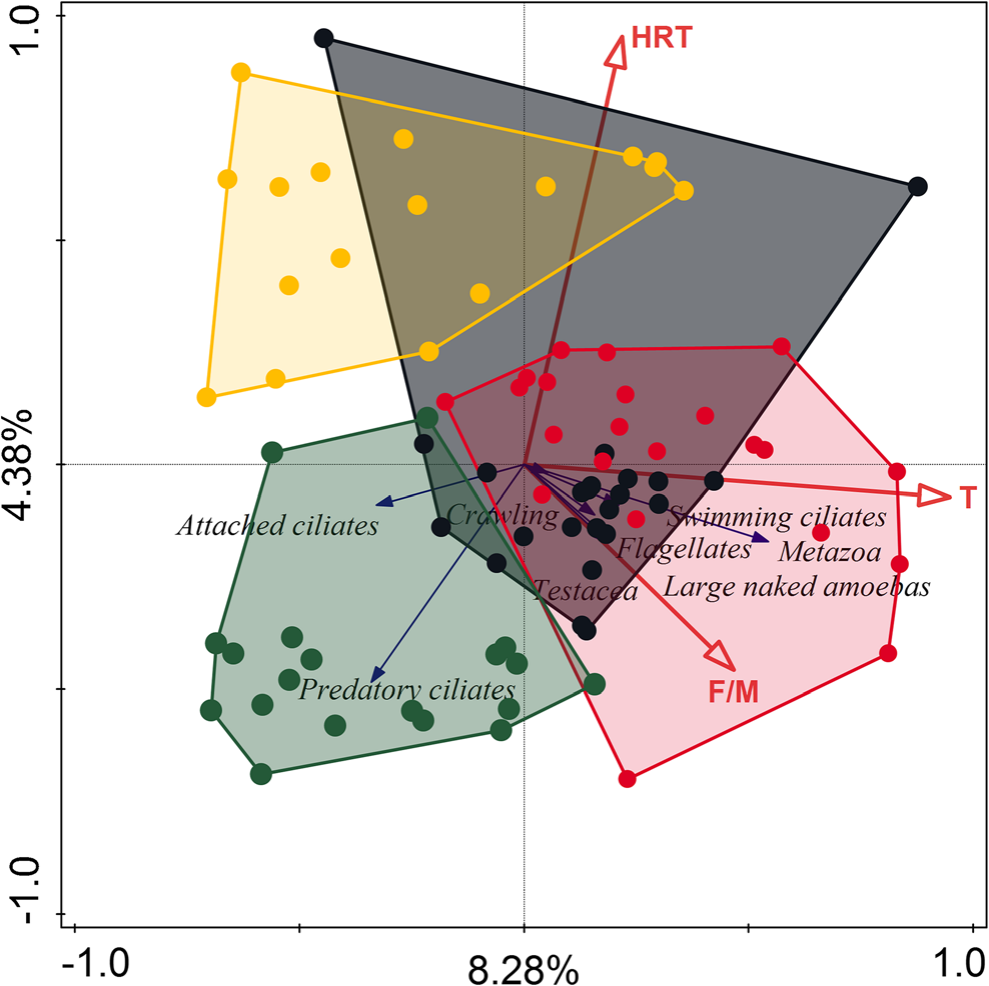
Triplot diagram from RDA summarizing the effects of process parameters descriptors upon functional/ecological groups in activated sludge. Dots represent WWTP: black—SK, red—NP, green—CH, and yellow—SI. About 8.28% (first axis) and 4.38% (second axis) of the variance in functional group composition were explained by process parameters descriptors

On RDA triplot diagram (Fig. 6) the first ordination axis is correlated mainly with the activated sludge temperature. The abundance of total metazoa tends to be larger at higher temperatures, and simultaneously abundance of attached ciliates tends to be larger at a lower temperature. The second ordination axis was more correlated with HRT. The density of predatory ciliates tends to be lower at higher HRT values. The density of testate amoebas, large naked amoebas, and flagellates tends to be higher at higher sludge load value. The effect of temperature, sludge load, and HRT on functional groups community was significant (*p* = 0.002), and both ordination axes in Fig. 6 explained 12.66% of the variance in functional groups composition.

As was mentioned earlier, WWTPs distinctly differ in values of operational parameters (Fig. 1; Tables 1 and 2) so it should be taken into account that the effects of process parameters upon species were also correlated with single treatment plant traits.

### Protozoa and metazoa community composition explained by process and operational parameters

**Fig. 7:**
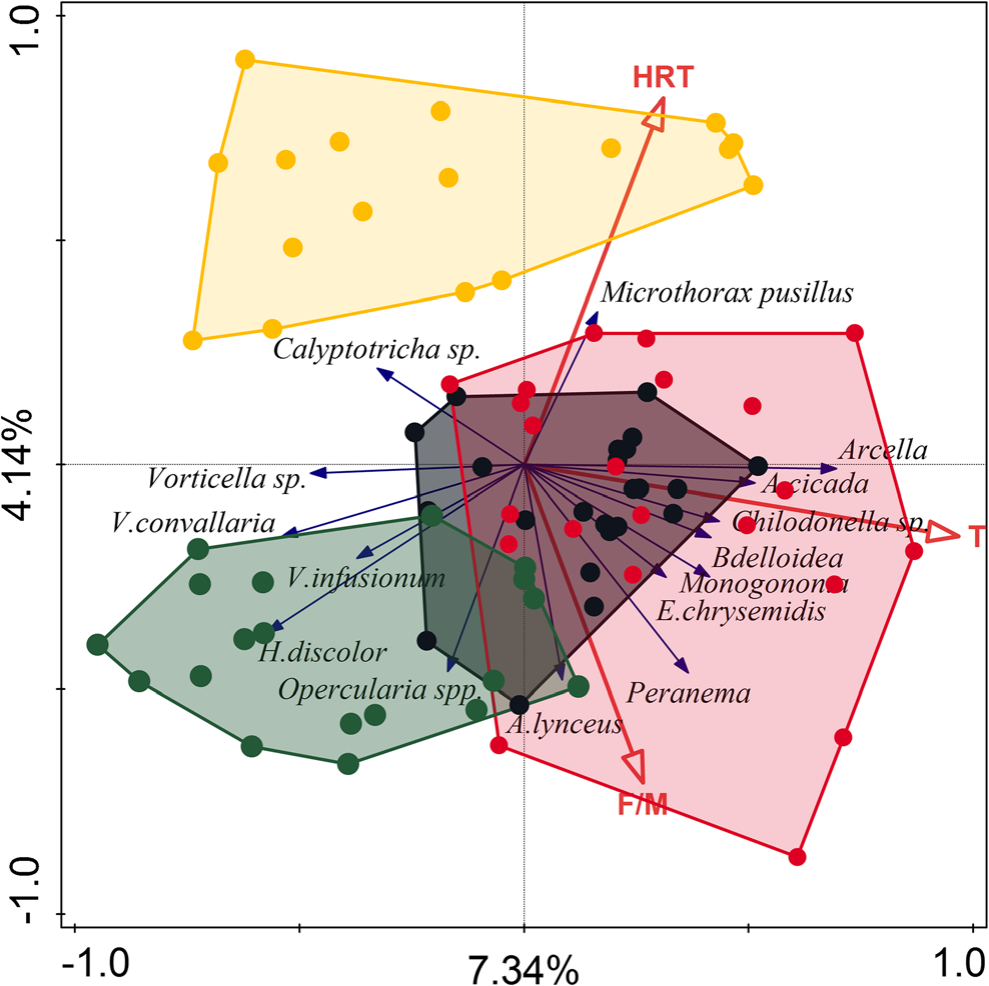
Triplot diagram from RDA summarizing the effects of process parameters descriptors upon protozoa and metazoa communities in activated sludge. Dots represent WWTP: black—SK, red—NP, green—CH, and yellow—SI, and 15 best fitted protozoa and metazoa representatives are shown. About 7.34% (first axis) and 4.14% (second axis) of the variance in microbial community composition were explained by process parameters descriptors.

On RDA triplot diagram (Fig. 7) the first ordination axis is correlated mainly with the activated sludge temperature. The abundance of testate amoeba—*Arcella* sp. and ciliates *A. cicada*, and *Chilodonella* sp.—tends to be higher at higher temperatures. On the other hand, the probability of *Vorticella* sp., *V. convallaria*, and *H. discolor* occurrence was higher at lower temperatures. The second ordination axis was more correlated with HRT. The density of the ciliate *M. pusillus* was higher at higher HRT values. In turn, the probability of the occurrence of attached ciliates *Opercularia* spp. was higher at lower HRT values. The effects of temperature, sludge load, and HRT on protozoa and metazoa community were significant (*p* = 0.002), and both ordination axes in Fig. 7 explained 11.48% of the variance in their community composition.

The SI WWTP microorganism community forms a separate cluster (yellow dots in Fig. 7) compared with other treatment plants in relation to investigated process parameters. As was mentioned earlier, WWTPs distinctly differ in values of operational parameters (Fig. 1; Tables 1 and 2) so it should be taken into account that the effects of process parameters descriptors upon protozoa and metazoa communities were also correlated with single treatment plant traits.

### Protozoa and metazoa community explained by SVI

**Fig. 8:**
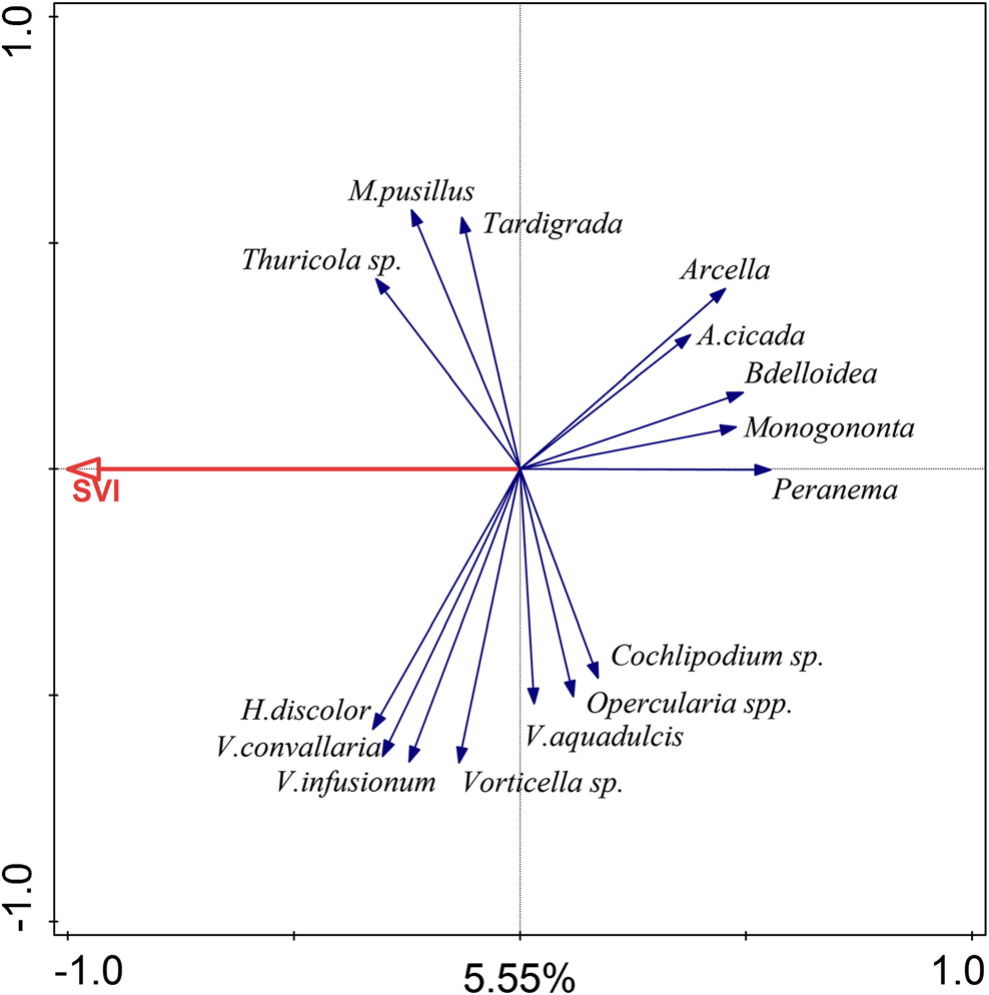
Biplot diagram from RDA analysis showing the effects of SVI upon protozoa and metazoa communities in activated sludge. The effect of SVI on protozoa and metazoa community was significant (*p* = 0.002). About 5.55% (first axis) of the variance in microbial community were explained

On RDA biplot diagram (Fig. 8) the abundance of flagellates *Peranema* sp. and both groups of rotifers: *Monogononta* and *Bdelloidea*, corresponded with the lower value of SVI.

### Protozoa and metazoa community composition explained by reduction rate of some pollution measures

**Fig. 9:**
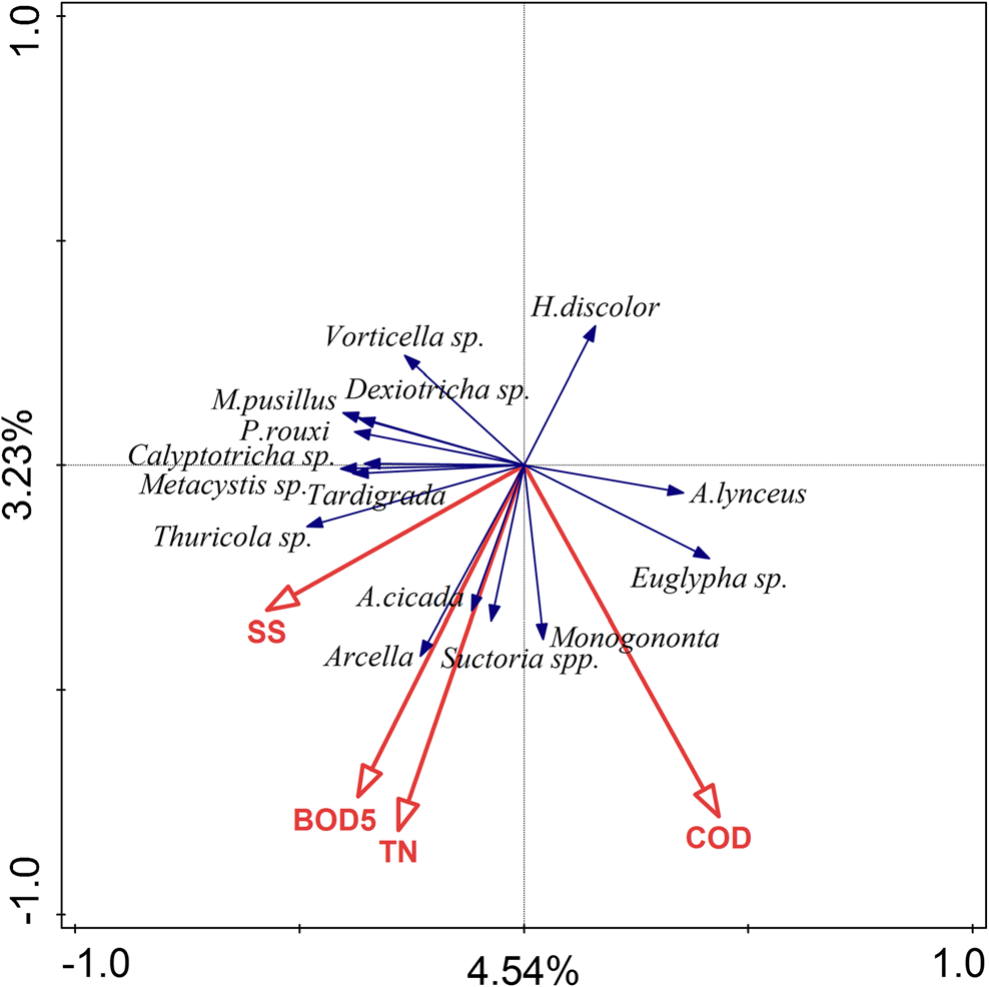
Biplot diagram from RDA analysis summarizing the effects of the reduction rate of pollution descriptors upon protozoa and metazoa communities in activated sludge. Fifteen best fitted protozoa and metazoa representatives are shown. About 4.54% (first axis) and 3.23% (second axis) of the variance in microbial community composition were explained by the reduction rate of pollution measures

In RDA analysis predictors, SS, BOD_5_, COD, and TN had insignificant (*p* = 0.086) effect on functional group composition (data not shown). On RDA biplot diagram (Fig. 9) the first ordination axis is weakly correlated with suspended solid reduction rate. The density of ciliates: *Thuricola* sp., *Metacystis* sp., *Plagiocampa rouxi*, and tardigrades, tend to be higher at higher suspended solid reduction rate. Testate *Arcell*a sp. abundance was strongly correlated with BOD_5_ reduction rate, while predatory ciliates *H. discolor* was strongly negatively correlated with BOD_5_ and TN reduction rate. Suctoria and *A. cicada* tend to be more abundant at higher BOD_5_ and total nitrogen reduction rate. The second ordination axis is weakly correlated with COD and the total nitrogen reduction rate. The probability of the occurrence of *Vorticella* sp. is lower when the values of COD reduction rate are higher. The higher reduction rate of BOD_5_ and TN corresponded with higher abundance of *Arcella* sp. Reduction rates significantly correlate with protozoa and metazoa community (*p* = 0.002), and both ordination axes explained 7.77% of the variance in their community composition.

## Discussion

Our results, similarly to Zornoza’s (2017) research, suggest that density and species composition of protozoa and metazoa in activated sludge depend on bioreactor configuration (volume and technology used). According to Zornoza, the temperature has more direct effect on variability in microbial communities than sludge age. On the other hand, the temperature is closely related to sludge age, because of common practice to increase the sludge age with the decrease of the nitrifying activity at the low temperatures in the bioreactors (Dymaczewski 2011).

In research conducted by Hu and co-workers (2013b) influent temperature and SVI showed the highest factorial loads in the first component axis in PCA exploring changes in protozoa and metazoa community. Results of our RDA analysis (Fig. 7) were similar, although instead of the temperature of influent, the temperature in a bioreactor of studied WWTPs was applied. Our study showed that the effect of temperature on protozoa and metazoa community was significant but the first ordination axis explained only 7.34% of the variance in community composition. Changes in SVI had a significant effect on protozoan and metazoan community but explained only 5.55% of the variance in species composition. It should be mentioned that occurrence and density of metazoa, especially rotifers, are correlated with temperature and SVI. Fiałkowska and Pajdak-Stós (2008) showed that rotifers *Lecane inermis* were able to consume and reduce the number of filamentous bacteria in activated sludge and are therefore able to reduce SVI value. These reductions strongly depend on temperature. With decreasing temperature, rotifer density decreases, and thus, rotifer pressure on filamentous bacteria weakens and as a consequence SVI values increases (Pajdak-Stós and Fiałkowska 2012). A similar and significant correlation between individual protozoa taxa and the temperature of influent, effluent, and activated sludge in aeration tanks was found by Ettl (2001). A strong temperature effect on the protozoa community structure could be explained by their correlation with the metabolic activity of ciliates (Laybourn and Finlay 1976). Likewise, Weisse et al. (2002) described an interaction between temperature and food concentration and their effect on the growth and production of planktonic protozoa. A recent study conducted by Wu et al. (2019) on 1200 activated sludge samples collected from 23 countries confirmed that temperature is a key factor influencing activated sludge bacterial community structure. Hai et al. (2014) showed that operational parameters: MLSS, SRT, HRT, and temperature, explained 19.9% of bacterial community variation. Fan et al. (2017) did not include the temperature as an operating factor but treated temperature as a separate factor and in their analysis temperature alone explained 9.20% of bacterial community variation. Likewise, Fredriksson et al. (2019) suggested that temperature and ethanol addition were the environmental parameters contributing the most to the temporal differences in bacterial community composition. As protozoa and metazoa are main bacterial feeders the changes in a bacterial community may directly affect protozoa and metazoan community. These results showed that factors independent of plant operators have the strongest effect on protozoa community in activated sludge of WWTPs.

Fluctuations in species composition and dominance structure in ciliate community during 1-year study were described by Ettl (2001) and Chen et al.(2004). Similarly to our study, the abundance and density of the different species were very variable, but simultaneously, the performance of all plants was fairly stable during the year of sampling. Thus, the researchers also did not find any consistent bioindicator species of process performance in protozoa and metazoa community.

In Tables 4 and 5 we gathered the results from studies conducted in different parts of the world. A comparison of results obtained by different authors also did not show any consistent relationships between ciliated protozoa species and process parameters. Basing on these comparative results it is hard to find a general pattern describing a relation between specific protozoa species and activated sludge process performance, even though for some species as *V. microstoma* or *A. cicada* some patterns were observed. Both ciliates *V. microstoma* and *A. cicada* tend to be generally negatively correlated with BOD_5_ and COD concentrations in the effluent (Table 4), whereas flagellates tend to be generally positively correlated with SVI, BOD, and SS concentration in effluent (Table 5).

**Table 4:** Value of correlation coefficients between ciliated protozoa species and process parameters in activated sludge investigated by others authors

Species	Effluent BOD	Effluent COD	Effluent SS	BOD removal	COD removal	SVI	Nitrification	Reference
*Aspidisca cicada*	− 0.233		− 0.123			0.02		Zhou (2006)
		− 0.087		− 0.064	0.064		− 0.452	Dubber and Gray (2011)
	− 0.34***	− 0.467*	− 0.462*					Drzewicki and Kulikowska (2011)
	− 0.791		− 0.82					Salvadó (1995)
	0.05	− 0.2	0.71***			− 0.26		dos Santos (2014)
	− 0.794**	− 0.833**						Papadimitriou (2007)
	− 0.18					0.13		Lee (2004)
*Aspidisca lynceus*		− 0.033		− 0.282	− 0.167		− 0.129	Dubber and Gray (2011)
*Acineria uncinata*		0.609*		− 0.309	− 0.336		− 0.345	Dubber and Gray (2011)
	− 0.01					0.22		Lee (2004)
	− 0.925		− 0.814					Salvadó (1995)
	0.892***	0.852***	0.665***					Drzewicki and Kulikowska (2011)
*Tritigmostoma cucullulus*		− 0.187			0.36		0.556	Dubber and Gray (2011)
	− 0.13					0.03		Lee (2004)
*Opercularia* spp.	0.358*		0.269*			− 0.19		Zhou (2006)
	0.17					− 0.13		Lee (2004)
	0.336*	− 0.108				0.11		Esteban (1991)
	0.21	0.03	− 0.08			0.06		dos Santos (2014)
	− 0.658**	− 0.692						Papadimitriou (2007)
				− 0.763***			− 0.597***	Madoni (2011)
*Vorticella convallaria*	− 0.402***		0.293*			− 0.103		Zhou (2006)
				0.609*	0.591		0.145	Dubber and Gray (2011)
	− 0.938		− 0.878					Salvadó (1995)
	− 0.614**	− 0.551						Papadimitriou (2007)
	0.65		0.857**	0.621*		− 0.56*		Ntougias (2011)
	0.1					− 0.11		Lee (2004)
*Vorticella microstoma*	− 0.1					− 0.26**		Lee (2004)
				− 0.679			− 0.504	Madoni (2011)
	− 0.608		− 0.572					Salvadó (1995)
	− 0.13	− 0.06						Papadimitriou (2007)
	0.21	− 0.21	− 0.37*			− 0.02		dos Santos (2014)
	− 0.342*	− 0.054				− 0.61**		Esteban (1991)
*Arcella* sp.	− 0.806**	− 0.895**	− 0.508**					Papadimitriou (2007)

*p < 0.05, **p < 0.01, ***p < 0.001

**Table 5:** Value of correlation coefficients between functional groups of protozoa and process parameters in activated sludge investigated by other authors

Functional group	Effluent BOD	Effluent COD	Effluent SS	BOD removal	SVI	Nitrification	Reference
Attached ciliates					0.66*		Hu (2012)
					0.85*		Hu (2012)
	− 0.34				− 0.03		Martin-Cereceda (1996)
	0.695**		0.891**	0.603**	− 0.63*		Ntougias (2011)
	− 0.247		− 0.2		0.15		Zhou (2008)
				0.432**		− 0.029	Madoni (2011)
Crawling ciliates				0.784***		0.62***	Madoni (2011)
	− 0.394**		− 0.283*		0.03		Zhou (2008)
	0.08				0.17		Martin-Cereceda (1996)
	0.17	− 0.17	0.77***		− 0.22		dos Santos (2014)
					− 0.26		Hu (2012)
					0.32		Hu (2012)
Swimming ciliates				− 0.829***		− 0.549***	Madoni (2011)
	0.078		0.073		0.27		Zhou (2008)
	0.52				0.03		Martin-Cereceda (1996)
	0.01		− 0.13		0.36		dos Santos (2014)
					0.28		Hu (2012)
					0.35		Hu (2012)
Flagellates				− 0.798***		− 0.596***	Madoni (2011)
	0.024		0.213		0.47***		Zhou (2008)
	0.43*	− 0.2	0.21		0.17		dos Santos (2014)
					0.48		Hu (2012)
					0.05		Hu (2012)
	0.67***	0.635***	0.62***				Drzewicki and Kulikowska (2011)
Testate amoebae				0.76***		0.912***	Madoni (2011)
	− 0.367***		− 0.281		− 0.52***		Zhou (2008)
	0.22*				0.09		Lee (2004)
	0.03	− 0.14	0.53**		− 0.29		dos Santos (2014)
					0.10		Hu (2012)
					− 0.28		Hu (2012)

*p < 0.05, **p < 0.01, ***p < 0.001

Zornoza (2017) showed that there were significant differences between bioreactors in environmental variables and seasonality, so it was impossible to construct one model of environmental interpretation which would help to explain population dynamics of protozoans, metazoans, and filamentous bacteria community. From the ecological point of view changes in the microbial community should be interpreted together with environmental variables in each bioreactor separately to develop models with better possibilities of predicting system functions (Zornoza 2017). Until now only a few researchers (Curds 1965, 1973, 1982; Ettl 2001; Madoni 2011) investigated ciliated protozoa in WWTP trying to explain changes in a microbial community. Numerous studies, e.g., Al-Shahwani and Horan (1991), Chen et al. (2004), Zhou et al*.* (2008), Hu et al. (2013a), and dos Santos et al*.* (2014), were limited to presenting a ciliated protozoa community composition and potential bioindication value of species without ecological interpretation and references to fluctuations of a microbial community.

For the purposes of this work, multivariate analysis of some process parameters as, e.g., temperature, sludge load, and HRT explained less than 12% of the variance in protozoa and metazoa community composition. This led to the conclusion that additional parameters should be included in the future analysis of activated sludge biocenosis, although in our study, we used all data available from WWTP operators. We agree with Zornoza (2017) who postulated that during exploration of the relationship between protozoan, metazoans, and environmental variables also the typology of these variables should be taken into account. For WWTP plant operators it is crucial to know which factors affecting the microbial community are under their control and which are not. Much earlier Salvadó and co-workers (1995) drew attention to limitations of assessment of effluent quality based on ciliates occurrences and densities. The presence of a particular ciliate species in activated sludge depends on several factors such as the composition of the influent, operational parameters, and the relations with the other species of the community. Similarly, Curds (1982) claimed that simple correlations between daily changes in BOD and the protozoa species structure should not be expected, because the structure of the protozoan population in sampling time reflects changes in physical, chemical, and biological environmental conditions over the past few days. Moreover, Curds and Cockburn (1970) underlined that their “indicator method” should not be used to predict effluent BOD concentration but should be treated as a tool to assess the general information about the efficiency of activated sludge performance.

Control of effluent quality on the base of microbial community composition is especially useful in the case of unexpected and undetermined toxic influents. The standard procedure of influent examination does not cover a plethora of toxic substances hampering aquatic organisms. The drastic decline of metazoans and protists diversity is most often a clear signal of disturbances caused by toxins, among them heavy metals (Madoni et al. 1996; Papadimitriou et al. 2007). It is highly probable that difficulties in the determination of clear patterns in the relation between protists’ composition and effluent quality are caused by such “noise” of toxic substances undetected by standard chemical analysis.

It is also worth to underline that in our research it was hard to discriminate bioindicators among protists as variance in performance of four examined WWTPs was very low. All investigated plants worked properly, without distinct perturbances, and with good effluent quality. All biological indicator systems should be regarded with caution since they oversimplify extremely complex ecological interactions (Curds and Cockburn 1970). However, we still do not have a cheaper and faster method of assessment of the potential environmental risk of WWTP effluent for rivers and lakes than microscopic evaluation of biodiversity of protozoa and metazoa inhabiting activated sludge.

## Conclusions

- The density and species composition of protozoa and metazoa in activated sludge depend mainly on bioreactor configuration (volume, technology used).
- For investigated treatment plants dominating species of protozoa and metazoa were defined.
- The joint effect of temperature, sludge load, and HRT on protozoa and metazoa community was significant.
- The effect of temperature on protozoa and metazoa community was the strongest but only slightly explained the variance in community composition.
- Changes in SVI had a significant effect on protozoa and metazoan community but explained only 5.55% of the variance in community composition.

## Electronic supplementary material

ESM 1 (DOCX 42 kb)
