# Supplementary material for: Multivariate analysis of activated sludge community in full-scale wastewater treatment plants
Source: Environ Sci Pollut Res Int. 2020 Sep 12;28(3):3579–89. doi: 10.1007/s11356-020-10684-5 (PMC7788020; doi:10.1007/s11356-020-10684-5)
Supplement: Supplementary file 1 — (DOCX 42 kb) [file 11356_2020_10684_MOESM1_ESM.docx]

Supplementary material

Table S1. Microorganisms from all distinguished groups found at least once in activated sludge of studied WWTPs.

| **Species** | **WWTP** | | | |
| --- | --- | --- | --- | --- |
|  | **SK** | **NP** | **CH** | **SI** |
| **Crawling ciliates** |  |  |  |  |
| *Aspidisca cicada* | + | + | + | + |
| *Aspidisca lynceus* | + | + | + | + |
| *Aspidisca turrita* | + | + | + | + |
| *Acineria uncinata* | + | + | + | + |
| *Euplotes affinis* | + | + | - | + |
| *Euplotes* sp. | + | + | + | + |
| *Chilodonella* sp*.* | + | + | + | + |
| *Cinetochilum margaritaceum* | - | - | - | + |
| *Cyrtophorida* spp. | + | + | + | + |
| *Drepanomonas revoluta* | + | - | - | - |
| *Microthorax pusillus* | + | - | - | + |
| *Trochilla minuta* | - | + | - | + |
| **Attached ciliates** |  |  |  |  |
| *Thuricola* sp. | + | + | + | + |
| *Epistylis plicatilis* | + | + | + | + |
| *Epistylis chrysemydis* | + | + | + | - |
| *Epistylis coronata* | + | + | + | + |
| *Epistylis* spp*.* | + | + | + | + |
| *Opercularia* spp*.* | + | + | + | + |
| *Vorticella infusionum* | + | + | + | + |
| *Vorticella microstoma* | + | - | + | + |
| *Vorticella convallaria* | + | + | + | + |
| *Vorticella aquadulcis* | + | + | + | + |
| *Vorticella* sp*.* | + | + | + | + |
| *Carchesium* sp. | + | + | + | + |
| *Calyptotricha* sp. | + | + | + | + |
| *Metacystis* sp*.* | + | + | - | + |
| **Swimming ciliates** |  |  |  |  |
| *Acineria incurvata* | - | - | + | - |
| *Dexiotricha* sp*.* | + | + | - | - |
| *Pseudocochnilembus pusilus* | + | + | - | - |
| *Cyclidium* sp*.* | - | + | - | - |
| **Predatory ciliates** |  |  |  |  |
| *Suctoria spp.* | + | + | + | + |
| *Holophrya* sp. | + | + | + | + |
| *Plagiocampa rouxi* | + | + | + | + |
| *Litonotus* sp. | + | + | - | - |
| **Testate amoebas** |  |  |  |  |
| *Arcella* sp*.* | + | + | + | + |
| *Euglypha* sp. | + | + | + | + |
| *Pyxidicula* sp. | + | + | - | + |
| *Cochlipodium* sp. | + | + | + | + |
| *Centropyxis* sp. | + | + | - | + |
| *Trinema* sp. | - | + | + | + |
|  |  |  |  |  |
| **Naked amoebae** | + | + | + | + |
| **Flagellates** | + | + | + | + |
| *Peranema* sp. | + | + | + | + |
| Small flagellates | + | + | + | + |
| **Rotifers** |  |  |  |  |
| Monogononta | + | + | + | + |
| Bdelloidea | + | + | + | + |
| **Nematoda** | + | + | + | + |
| **Tardigrada** | + | - | - | + |
| **Gastrotricha** | - | + | - | - |
